# At-risk youth receive similar benefits from equine- assisted psychotherapy and traditional psychotherapy; an applied analysis

**DOI:** 10.3389/fpsyt.2025.1518783

**Published:** 2025-04-29

**Authors:** Cindy E. McCrea, Grace Tibbets, Levi W. Smith, Cynthia G. Campbell

**Affiliations:** Department of Psychological Sciences, Boise State University, Boise, ID, United States

**Keywords:** equine-assisted psychotherapy (EAP), well-being, adolescents, program evaluation, community-based

## Abstract

**Introduction:**

Equine-assisted psychotherapy (EAP) is a promising modality for the treatment of emotional difficulties in youth. Few studies have compared the benefits of EAP to those of traditional psychotherapy for at-risk youth in community-based settings.

**Method:**

We compare the effectiveness of individually administered EAP and traditional psychotherapy (TP) for improving adolescent mental health using data from a community-based participatory research partnership. Adolescent clients who were referred to a community-based non-profit agency for services related to emotional and behavioral difficulties comprised the sample (N = 94, mean age at intake was 14.33 years). We compared the improvement in mental health outcomes between intake and follow-up for participants who received weekly, individual TP (N = 65) with those who received weekly, individual EAGALA-certified EAP (N = 29). Licensed mental health professionals administered treatments (mean treatment period of 241 days).

**Results:**

On average, clients’ global psychological, social, and emotional wellness scores on the Mental Health Continuum improved by 18-23%. Clients’ resilience, self-efficacy, social and emotional skills, perceptions of hope, and cognitive reappraisal skills also improved significantly (12% to 28%) with one exception; average emotion suppression scores did not change across the treatment period in either group (*p* = .77). Mixed linear models revealed that clients receiving EAP and TP experienced similar levels of improvement in all dimensions.

**Discussion:**

These quasi-experimental data confirm that community-based non-profit programs that offer psychotherapy benefit at-risk youth and indicate that EAP and TP may provide similar benefits to struggling youth.

## 1 Introduction

Equine-assisted psychotherapy (EAP) has gained attention in recent years as a possible modality for promoting psychological, emotional, and social well-being among children and adolescents (1, 2). Theoretically, there is good reason to think that EAP might benefit struggling youth. Since equines are sensitive to nonverbal behavior and provide immediate feedback to handlers, learning to communicate with and guide a horse can facilitate increases in self-awareness and self-regulation (3, 4). Others posit that self-esteem and self-image are strengthened by grooming and caring for horses because participants adopt the role of responsible caretakers rather than patients when engaging in this type of therapy (5). EAP may be particularly helpful for youth with a history of trauma; While it can be challenging for these children to form healthy therapeutic relationships (6–8), engaging with animals provides a potential pathway for circumventing this barrier (3, 9, 10). Finally, working directly with equines may produce calming autonomic system responses; indeed, the release of oxytocin and inhibition of cortisol have been observed after equine interactions (11, 12).

Many studies indicate that equine-assisted programs are beneficial for youth at risk of poor outcomes, including those who have experienced abuse, exhibit behavioral problems, or meet the criteria for depression, anxiety, or PTSD (13–17). Improvements in self-efficacy and self-esteem (18, 19), and improvements in coping skills and emotion regulation have been observed following equine interactions in at-risk youth (20, 21). EAP may also improve social functioning and interpersonal relationships. For example, among adolescents with mental and behavioral concerns, weekly group equine-assisted learning sessions contributed to significant reductions in anti-social behavior and improved socialization skills (22). Clinically meaningful reductions in depression and anxiety have also been observed following EAP in this population (23–25).

However, very few EAP protocols have included a comparison group, limiting our ability to understand the value of EAP relative to traditional methods for treating psychosocial concerns in at-risk youth. The majority of recently published findings for equine interventions used to improve youth emotional well-being did not include a control or comparison group of any kind (10, 17, 18, 22, 23, 26–34). Those that do offer a comparison group usually compare equine-assisted therapy with a group of waitlisted control participants (13, 16, 20, 25, 35–37). The lack of controlled studies limits our understanding of the utility of equine-assisted therapies relative to standard psychotherapy.

Only a small set of recent studies directly compare the psychosocial benefits of EAP with standard treatment for improving emotional well-being in samples of youth. Authors of a recent meta-analysis of equine-assisted interventions for youth found just 13 studies published in the last 25 years that included credible comparison treatments (2). While their results revealed significant improvements in externalizing, and internalizing behaviors and efficacy (but not depression or self-esteem) following equine-assisted therapies, the team cited the need for additional reports with larger samples, randomization, or at least tests for equivalence between groups at baseline, and comparison groups with credible treatments instead of waitlisted controls.

While EAP is increasingly understood to be an effective therapeutic technique, it is less clear how the efficacy of such therapy compares to standard outpatient psychotherapy for youth at general risk of poor outcomes. Further research on its effectiveness is needed to determine if investments in and referrals for EAP are well placed. Thus, we evaluated the efficacy of EAP compared to TP for improving psychosocial outcomes in youth referred for psychotherapy.

## 2 Materials and methods

### 2.1 Participants

Clients receiving services from a community-based non-profit agency focused on improving the life trajectories of at-risk youth comprised the sample. Outpatient clients engaged in individual therapies who completed both the intake survey and a follow-up or discharge survey between July 1, 2019 and June 30, 2022 are included in this analysis (N = 94; *M* age at intake was 14.33 years; *M* length of service was 241 days). Outcomes for clients who never engaged in EAP but received TP (TP group; N = 65, mean treatment length = 253.6 days) were compared to outcomes of clients who received EAP as the primary element of their treatment plan (EAP group; N = 29, mean treatment length = 214.7 days). The majority of clients in both groups were female (see **Table 1**).

**Table 1:** Sample characteristics and intake values by treatment group.

	EAP	TP	*p*
	M ± SE	M ± SE	
Length of Service (*days*)	214.7 ± 43.18	253.6 ± 24.18	.40
Age at Intake (*years*)	13.38 ± 0.41	14.76 ± 0.35	.02
Intake Scores			
Child Hope Scale	56.86 ± 4.84	54.61 ± 2.64	.66
Agency	53.75 ± 5.10	55.61 ± 2.92	.74
Pathways	59.88 ± 5.15	54.26 ± 2.67	.34
ERQCA			
Cognitive Reappraisal	62.41 ± 4.45	50.80 ± 2.70	.02
Emotion Suppression	50.96 ± 4.82	59.42 ± 2.72	.10
Youth Thrive Survey			
Youth Resilience	58.47 ± 4.73	51.13 ± 2.61	.15
C S-E Competence	65.36 ± 3.49	63.35 ± 1.77	.61
Mental Health Continuum			
Emotional W-B	57.15 ± 5.13	57.85 ± 3.73	.91
Psychological W-B	66.25 ± 4.12	57.54 ± 2.91	.09
Social W-B	55.94 ± 3.92	48.12 ± 3.50	.18
Community Empowerment			
Self-Efficacy	50.72 ± 5.69	46.14 ± 3.41	.47

### 2.2 Procedures

The non-profit partner for this project serves youth with a wide range of concerns from emotional distress, and behavioral concerns to academic risk and family dysfunction. Youth were referred to the program from a variety of sources including social workers, teachers, school counselors, juvenile justice programs, parents or self-identification. Treatment plans were determined in consultation with the client, family, and other invested partners (i.e., probation officer, foster parent, special education teacher). As per standard procedure, the clinical program manager reviewed each client’s information and guided treatment modality decisions based on fit. Clients with known barriers to TP (ie., may have trouble sitting for a typical session, ADHD diagnosis) or who had not been responsive to TP in the past were more likely to be referred to EAP. Procedural and legal limitations also influenced modality decisions. For example, when applicable, sex offender-specific treatments had to be completed before engagement in equine therapy. Clients who were actively suicidal, homicidal or experiencing psychosis were not eligible for EAP. Capacity caps limited the number of clients who could receive services at any given time and the capacity for EAP was lower than TP. Thus, the number of clients in each group differed expectedly. The length of treatment varied widely for clients and was determined by client needs. When the client, family, and therapist team agreed that treatment plan goals had been met, a final celebration session was held at discharge. For clients that had transportation issues, case managers worked to set up transportation for clients or provide gas cards to families in need. Although each client in this analysis was assigned to receive weekly EAP or TP as their primary treatment plan, clients may have received other program interventions, including specialized treatment for specific concerns (i.e., eating disorder treatment, substance abuse treatment). Individualized EAP or TP sessions may have been adapted to complement those treatments. Complementary therapies may have included group or family-focused sessions.

### 2.3 Intervention

#### 2.3.1 Equine-assisted psychotherapy

Clients in this group received EAP sessions during all or part of their treatment plan. Clients typically engaged in weekly one-hour sessions patterned after the Equine Assisted Growth and Learning Association (EAGALA) model of therapy. EAP typically lasts 4-6 months. The therapy team was composed of an equine, a licensed clinical social worker (LCSW) or other mental health clinician, and an equine specialist. At least one, but often both personnel were EAGALA certified. Horses of all backgrounds and temperaments were engaged; only safety concerns (i.e., history of biting or kicking) excluded a horse from joining a team. The EAGALA method has been described in detail elsewhere (EAGALA.org). The essential elements of the practice are client engagement with an equine, which may be as simple as observation or as complex as directing movements (but never riding). Verbal processing with an LCSW or licensed therapist also occurs. A client may interact with just one or several different horses during early sessions. Once a horse comes to represent a meaningful construct in a session (i.e., “This horse is a bully, just like those bullies at school.” or “This horse is angry like me.”), then the horse is repeatedly engaged for subsequent sessions. The individual EAP sessions are directed organically toward clients’ goals and needs. The therapy arena is indoors (though not heated), allowing for nearly year-round EAP delivery.

#### 2.3.2 Traditional psychotherapy

Clients in the TP group received services from the same community-based organization during the same years but never engaged in EAP. Instead, they engaged in TP with LCSWs or licensed therapists at established outpatient clinics. The typical treatment program involved weekly one-hour sessions with the provider. Therapeutic approaches included Cognitive Behavioral Therapy (CBT), Dialectical Behavioral Therapy (DBT), Trauma-focused Cognitive Behavioral Therapy (TF-CBT), and Eye Movement Desensitization and Reprocessing (EMDR). The individual sessions are directed organically toward clients’ goals and needs.

#### 2.3.3 Data collection protocol

Clients are routinely asked to complete psychosocial assessments at intake and discharge. Additional assessments are requested at 90, 120 and 180 days of service if discharge has not already taken place. For this analysis, only clients with both a true intake assessment (i.e., within 2 weeks of their first session) and a follow-up or discharge assessment are included. When multiple follow-up assessments were completed, the most recent assessment was used. Clients responded to survey questions by moving a slider on a 0 to 100-point scale.

### 2.4 Measures

The Children’s Hope Scale is a six-item self-report index for children ages 8-16 that measures perceived levels of agency toward goals (38). The psychometrics of this scale are well-established; the median Cronbach’s alpha score from 7 youth samples demonstrates good internal consistency (ɑ = 0.77) and test-retest reliability is also reasonably robust (r = 0.71; 38, 39).

The Youth Resilience Subscale from the Youth Thrive™ Survey is a 10-item subscale targeted at youth and young adults between the ages of 12 and 26 (40, 41). The evaluation assesses the youth’s ability to rise to life’s challenges, including how they handle daily stress and overcome trauma or adversity. The scale has high internal consistency (ɑ = 0.88, 42). The 16-item Cognitive and Social-Emotional Competence Subscale (CSEC) is used to evaluate the knowledge, attitudes, and skills youth display related to navigating their identity and interactions with others (40)). The internal consistency of this subscale is also high (ɑ = 0.84; 42). A confirmatory factor analysis of the full survey also revealed expected levels of discriminant validity between the subscales (42).

The Emotion Regulation Questionnaire for Children and Adolescents is a ten-item scale that assesses a child’s ability to re-appraise negative emotions and their tendency to suppress emotions (43). The Expressive Suppression and Cognitive Reappraisal Subscales, with 4 and 6 questions respectively, measure separate facets of how emotions are regulated. The ES subscale contained 1 item assessing suppression of positive emotions, 1 of negative emotions, and 2 general suppression items. High test-retest reliability has been demonstrated for both scales (ɑ = 0.75 and 0.83 respectively) and acceptable convergent validity between each subscale and other established measures of emotional functioning has also been reported (44).

The Mental Health Continuum-Short Form (MHC-SF) is a 14-item survey consisting of 3 subscales targeted at assessing different facets of well-being (45). The Emotional Well-Being subscale contains 3 items that focus on the participant’s emotional well-being within the past month. The Social Well-Being subscale is comprised of 5 items that focus on the participant’s social well-being, how they view society as a whole, and their place within it. In the Psychological Well-Being subscale, 6 items are focused on the participant’s overall psychological well-being, including their outlook on life and their recent emotional status (46). The MHC-SF demonstrates acceptable internal reliability for each subscale and as a whole (ɑ = 0.74 - 0.83) and correlates well with other measures of emotional, social and psychological well-being (47).

The Self-Efficacy Subscale from the Individual Community-Related Empowerment survey was used to assess levels of empowerment, or the degree to which an individual views themselves as having the power to make improvements and contributions within their community (48). This subscale is psychometrically robust with a content validity coefficient of 0.98 and Cronbach’s alpha of.883 for internal consistency (48).

### 2.5 Statistical analysis

Independent samples t-tests were used to evaluate equivalence between the two treatment groups at baseline (see **Table 1**). To assess the overall effectiveness of the program, paired t-tests were used to compare intake scores to discharge scores for each outcome for the entire sample (see **Table 2**). The data met the assumptions required for these tests.

**Table 2:** Psychosocial functioning before and after therapeutic services.

Outcome	N	Intake	Follow-up	Change		*p*
		M ± SE	M ± SE		%	
Child Hope Scale	94	55.30 ± 2.4	69.13 ± 2.10	13.83	25.01%	<.0001
Agency	94	55.03 ± 2.54	66.37 ± 2.33	11.34	20.61%	<.0001
Pathways	93	56.01 ± 2.44	72.02 ± 2.05	16.01	28.60%	<.0001
ERQCA						
Cognitive Reappraisal	91	54.37 ± 2.4	66.13 ± 2.03	11.76	21.63%	<.0001
Emotion Suppression	92	56.75 ± 2.42	52.45 ± 2.52	-4.3	7.5%	.26
Youth Thrive Survey						
Youth Resilience	72	53.57 ± 2.4	67.01 ± 2.00	13.54	25.32%	<.0001
C S-E Competence	73	64.00 ± 1.63	71.92 ± 1.6	7.92	12.40%	<.0001
Mental Health Continuum						
Emotional WB	71	57.62 ± 3.0	71.34 ± 2.26	13.72	23.81%	<.0001
Psychological WB	69	60.32 ± 2.41	71.73 ± 1.79	11.41	18.92%	<.0001
Social WB	91	50.68 ± 2.7	59.71 ± 2.26	9.03	17.82%	.01
Community Empowerment						
Self-Efficacy	88	47.60 ± 2.93	57.90 ± 2.70	10.30	21.64%	<.0001

We used mixed linear models in SAS version 9.4 to test the fixed effects of treatment type (TP vs. EAP) on the magnitude of change in each outcome score. Change scores for each client and each outcome type were calculated as follow-up score minus baseline score; thus larger positive numbers reflect larger improvements than smaller numbers except in the case of emotion suppression where reductions are favorable. We included the covariates sex, age at intake, length of service and, to account for baseline differences, the intake score of the scale of interest in each model. Models were optimized by removing non-significant covariates when their removal improved model fit statistics as indicated by lower values on the Bayesian Information Criterion. All models passed a final check for normality of the residuals. **Table 3** presents least-square means and standard error values from these models.

**Table 3:** Adjusted mean change in outcome scores by therapy type.

Outcome	Equine-assisted Psychotherapy	Traditional Psychotherapy	*p*
	M ± SE	M ± SE	
Child Hope Scale	14.98 ± 3.7	14.15 ± 2.3	.85
Agency	13.45 ± 4.12	11.18 ± 2.6	.65
Pathways	16.05 ± 3.74	17.02 ± 2.34	.83
ERQCA			
Cognitive Reappraisal	13.14 ± 4.0	13.11 ± 2.43	.99
Emotion Suppression	-3.16 ± 5.01	-3.99 ± 3.17	.89
Youth Thrive Survey			
Youth Resilience	13.52 ± 4.0	12.32 ± 2.51	.80
C S-E Competence	8.76 ± 3.0	6.46 ± 1.9	.52
Community Empowerment			
Self-Efficacy	13.24 ± 4.65	11.53 ± 3.01	.76

## 3 Results

Global psychological, social and emotional well-being scores improved from baseline to follow-up by 18-23% among all participants (see paired-t test results in **Table 2**). The adaptive perspectives and cognitive skills measured also improved significantly (12% to 28%) with one exception; average emotion suppression scores did not change across the treatment period (*p* = .77).

### 3.1 Effects of EAP v.s. TP

There were some differences between groups at baseline; EAP clients were significantly younger than TP clients at intake (*M* = 13.4 yrs vs. *M* = 14.8 yrs, *p* = .02). They also exhibited greater baseline cognitive reappraisal skills than TP clients (*p* = .02; see **Table 1**).

**Figure 1:**
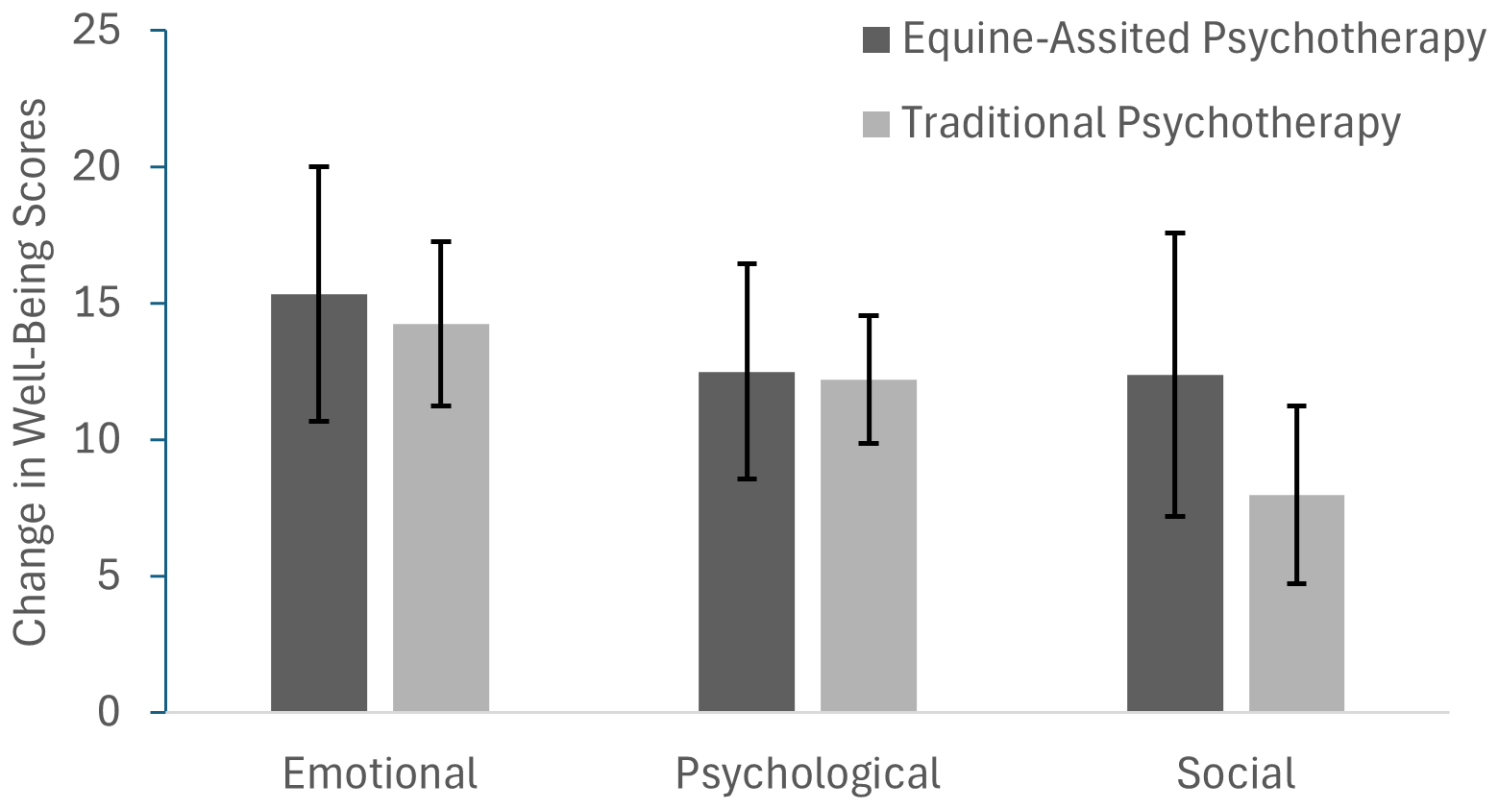
Change in well-being scores by treatment group. Clients in the EAP group (equine-assisted psychotherapy therapy) and the traditional psychotherapy group (TP) experienced statistically similar improvements in emotional, psychological and social well-being.

Clients receiving EAP and TP experienced statistically similar levels of positive change or improvement in all three areas of global wellness; there were no significant differences between the improvements in emotional, social or psychological well-being scores between the two groups (see **Figure 1**). Similarly, clients in both groups experienced similar improvements in the cognitive skills and adaptive perspectives assessed (**Table 3**). No change in emotion suppression was detected in either group.

## 4 Discussion

As expected, adolescent clients experienced significant psychosocial benefits from receiving community-based non-profit therapeutic services. On average, clients experienced 18-23% gains in self-reported ratings of psychological, social, and emotional well-being after engaging in services (**Figure 1**). Notably, clients self-reported that their hope and resilience scores, strong predictors of success-oriented behaviors and positive adjustment in youth (49, 50), improved following services. A 22% increase in cognitive reappraisal skills, known to be associated with enhanced well-being and prosocial behavior (51–53), was also observed following treatment. These data are consistent with a large body of work that demonstrates that psychotherapy is effective at improving the well-being of adolescent clients (54) and that community-based psychotherapy programming is effective (55).

Improvements in all psychosocial outcomes were similar for those engaged in EAP and TP. Psychosocial improvements following EAP might be the result of confidence and emotion management developed in the horse arena, crossing over into other areas of life (33, 56–58). While these data cannot corroborate or disprove mechanistic theories, they do indicate that using EAP as the primary treatment mode provides similar benefits for struggling youth as traditional psychotherapy. Notably, in this nonrandom design, a history of nonresponsiveness to past TP was one criterion for placing clients in the EAP group. Despite this bias, rates of improvement during treatment were similar between the groups. Likewise, the EAP group was slightly younger and had slightly better cognitive reappraisal scores than the TP group at baseline. Despite these baseline differences, rates of improvement were similar between the two groups.

Notably, clients in neither group experienced desired reductions in emotion suppression. Neuroimaging studies show that emotion suppression is cognitively taxing and activates stress physiology that can negatively influence immune function and physical health (59). Since high levels of emotion suppression create significant psychological distress (60), emotion regulation strategies that reduce suppression serve to promote resilience through difficult childhood experiences (61). Congruently, replacing maladaptive emotion regulation strategies with adaptive ones is encouraged as a central goal of youth treatment programs that seek to promote resilience (62). Given that we observed improvements in outcomes that are known to covary with emotion suppression (resilience, cognitive reappraisal), and that others have reported suppression to be modifiable in youth (63) the lack of change in this area is difficult to interpret.

Notably, most research connecting suppression to negative outcomes refers to the suppression of negative emotions. The scale used herein included questions related to suppressing both negative and positive emotions, the latter of which may not be problematic (64). In fact, from an adolescent perspective, the ability to suppress positive emotions (ie., laughter during a school session) may be beneficial and related to improved social awareness, engagement, and desirable emotion regulation (65). Indeed, recent factor analyses of an expanded version of the scale provided estimates that support separating the measurement of suppression for positively and negatively valenced emotions and indicate that they differentially relate to mental health (66). Thus the measure utilized in our study, while psychometrically sound, may not be an optimal indicator of psychosocial health. Additionally, any reductions in negative emotion suppression may have been counterbalanced by potential improvements in adaptive positive suppression.

Generalization of these results are limited by varied levels of client engagement and the use of non-random assignment. This analysis was limited to clients who completed both intake and follow-up surveys within the study period. Clients who failed to complete data collection instruments may have differed from those who did. For example, extreme distress at the first visit may reduce the likelihood of clients completing intake data. The number of clients that the organization served but failed to complete the surveys necessary to be represented in this sample is unknown. Therefore, this data does not represent how the average client in the program fared; rather, it represents how clients who completed both survey instruments responded to treatment. Second, instead of using random assignment, the clinical manager made treatment decisions based on need and fit, an approach that, while methodologically limiting, was ethically aligned with this organization’s mission.

As expected with non-random procedures, the groups differed systematically in some ways. The EAP group was younger and had better cognitive reappraisal skills at program entry than the TP group. They may have also differed in other, unassessed ways. For example, given that EAP and TP are offered at different sites, the proximity of the client’s residence to a specific site could have influenced retention (and therefore inclusion in this analysis). Clients with greater parental support or high socioeconomic status may have been likely to successfully adopt a treatment plan that required regularly traveling to the more rural EAP center (note that if staff were aware of cost-related transportation barriers, gas cards were provided). Neither the client diagnosis nor the concurrent use of complementary treatments were documented for this study. These characteristics may have differed by group. Despite the few known and possible unknown baseline differences, the rate of psychosocial improvement in the two groups was the same. That is, clients in each group improved at the same rate regardless of their starting point. This ecologically valid method of treatment assignment resulted in similar improvements across both groups. However, generalizations should be made cautiously since non-random assignment may be associated with inflated effect sizes (67).

Additional limitations include the self-reported nature of the data and the lack of data related to dropout rates, unknown diagnoses and cultural diversity. Subjective differences between clients in perceived improvement and well-being may differ greatly, and the collected data may suggest perceptual improvements rather than empirical improvements to psychosocial well-being. Future studies may seek to incorporate data from clinician assessments, although this method of assessment is costly and time-consuming. To account for this obstacle, data collection from other informants such as parents or teachers may provide sufficient information. Qualitative and mixed-method designs should be employed in future studies to mitigate the subjectivity of client perception (23). Specific diagnoses or reasons for seeking treatment were not available to the research team. As a result, highly desirable subgroup analyses to determine potential therapeutic targets were not possible. Given the demographics of the communities this organization serves, the sample was likely predominantly Caucasian. We did not have the data necessary to assess differences in outcomes based on race or ethnicity, though such work is important (14).

These data indicate that community-based non-profit programs that offer therapy delivered by licensed therapists benefit at-risk youth. We provide evidence that youth with psychosocial challenges may receive similar benefits from treatment plans that primarily focus on consistently delivered, individualized EAP sessions as clients whose treatment plans are structured around individualized TP.

## Data Availability

The data analyzed in this study is subject to the following licenses/restrictions: This is a private dataset which contains sensitive data from minors. It is only available to IRB approved study personnel. Requests to access these datasets should be directed to cindymccrea@boisestate.edu.
